# Beneficial Microorganisms to Control the Gray Mold of Grapevine: From Screening to Mechanisms

**DOI:** 10.3390/microorganisms9071386

**Published:** 2021-06-25

**Authors:** Zakaria Amarouchi, Qassim Esmaeel, Lisa Sanchez, Cédric Jacquard, Majida Hafidi, Nathalie Vaillant-Gaveau, Essaid Ait Barka

**Affiliations:** 1 Université de Reims Champagne-Ardenne, RIBP EA4707 USC INRAE 1488, SFR Condorcet FR CNRS 3417, 51100 Reims, France; zakariaamarouchi@gmail.com (Z.A.); qassim.esmaeel@univ-reims.fr (Q.E.); lisa.sanchez@univ-reims.fr (L.S.); cedric.jacquard@univ-reims.fr (C.J.); nathalie.gaveau@univ-reims.fr (N.V.-G.); 2 Laboratoire de Biotechnologie Végétale et Valorisation des Bio-Ressources, Faculté des Sciences, Université Moulay Ismail, Meknès B.P. 11201, Morocco; hafidimaj@yahoo.fr

**Keywords:** gray mold, biocontrol, grapevine

## Abstract

In many vineyards around the world, *Botrytis cinerea* (*B. cinerea*) causes one of the most serious diseases of aerial grapevine (*Vitis vinifera L*.) organs. The control of the disease relies mainly on the use of chemical products whose use is increasingly challenged. To develop new sustainable methods to better resist *B. cinerea*, beneficial bacteria were isolated from vineyard soil. Once screened based on their antimicrobial effect through an in vivo test, two bacterial strains, S3 and S6, were able to restrict the development of the pathogen and significantly reduced the Botrytis-related necrosis. The photosynthesis analysis showed that the antagonistic strains also prevent grapevines from considerable irreversible PSII photo-inhibition four days after infection with *B. cinerea*. The *16S rRNA* gene sequences of S3 exhibited 100% similarity to *Bacillus velezensis*, whereas S6 had 98.5% similarity to *Enterobacter cloacae*. On the other hand, the in silico analysis of the whole genome of isolated strains has revealed the presence of “biocontrol-related” genes supporting their plant growth and biocontrol activities. The study concludes that those bacteria could be potentially useful as a suitable biocontrol agent in harvested grapevine.

## 1. Introduction

Pathogens cause a devastating impact on crops varying from economic hardship to poisoning of food supplies (such as ergotism) and horrendous famines such as the Irish potato famine that lasted from 1845 to 1852. After their assaults, pathogens might trigger substantial changes to the host physiology, which can occur directly by secreting toxins and lytic enzymes or indirectly through inducing host responses stimulated by the pathogen. Among the significant physiological processes, the photosynthesis is the principal process affected by foliar diseases [1]. The photosynthesis decline might be proportional to decrease in green leafy tissue. Furthermore, the decline in photosynthesis, infections can trigger other physiological changes such as limited water use efficiency, which in turn, excessive water restriction may further induce a lower rate of photosynthesis (as reviewd in [2]). The pathogen might also impact the net carbon assimilation rate by enhancing the leaf respiration, which is requested to supply the demand initiated by the accelerated cells host metabolic activity [3]. The photosynthesis decreases through the infection process as a result of repression of photosynthetic gene expression [4,5]. Despite various struggles to decipher mechanisms by which pathogens can disturb photosynthetic capacity, current knowledges of the subject remain far from inclusive.

Plants do not have a circulatory system and adaptive immune system like animals. To block pathogen progress, plants have evolved a two-layered innate immune system. The first line of plants defense is accomplished via a set of defined receptors, namely pattern recognition receptors (PRRs), which able to identify conserved microbe-associated molecular patterns (MAMPs) [6]. Following MAMPs recognition, MAMP-triggered immunity (MTI) primary defense responses are triggered including mitogen-activated protein kinase (MAPK) phosphorylation cascades, cell wall alterations, callose deposition, defense genes expression, and defense-related proteins accumulation [7,8].

When perceived by intracellular immune receptors, pathogen effectors trigger the effector-triggered immunity (ETI; [8]). The second stage of perception uses the recognition of microbial effectors, the virulence factors that suppress MTI to initiate effector-triggered immunity (ETI), triggering a cascade of complex signaling events, leading to suppression of pathogen assaults.

*Botrytis cinerea* is one of the highest broadly studied necrotrophic fungal pathogens. *B. cinerea* has no apparent host specificity infecting therefore, more than 1000 plant species [9]. The gray mold (GM) caused by *B. cinerea* has a devastating impact on various economically important crops, including grape, strawberry, and tomato [10] with annual economic losses exceeding USD 10 to 100 billion worldwide [9,11,12].

Several disease controlling approaches have been implemented in the past and present to control *B. cinerea*. Currently, pesticides remain the main method used to fight the pathogen, and in some instances, the only option, involving significant financial costs. Until lately, the use of chemical fungicides to protect plant was thought to be fairly safe. Nevertheless, more than ever before, chemical fungicides use faces multiple challenges namely the development of resistance to fungicides resulting in the decline or even failure of control effect [13,14,15,16], increased consumers desire of food free of pesticide residues [17], enhanced concern regarding environmental pollution, and stricter regulatory policies are being imposed on the use of synthetic chemical fungicides [17,18].

To overcome the difficulties previously stated, it is urgent to search for alternative, effective, and eco-friendly strategy of disease control [19,20,21]. Thus, in recent years, the use of microbes as a biocontrol agent is gaining interest in agriculture [22].

Plant growth promoting rhizobacteria (PGPR) are bacteria that inhabit the rhizosphere and can improve the extent or quality of plant growth directly and or indirectly. The direct promotion by PGPR involves either delivering plant with a plant growth promoting substances or helping plants to mobilize and acquire nutrients from the rhizosphere. The indirect effect occurs when PGPR prevent the harmful effect of pathogens. In the last few decades, bacteria including species of *Arthobacter*, *Azospirillum*, *Azotobacter*, *Bacillus*, *Burkholderia*, *Enterobacter*, *Klebsiella*, *Pseudomonas*, and *Serratia* have reported to enhance plant fitness.

Various mechanisms for antagonism have been implicated, such as competition for nutrients and space, secretion of cell wall degrading enzymes, siderophores [23,24], parasitism of the pathogen, biofilm formation, induction of host defenses via production of various pathogenesis related proteins (PR) [25], and the involvement of reactive oxygen species (ROS) in the defense response are responsible for their antagonistic activity [18]. PR proteins regulated resistance in response to *B. cinerea* in grapes. Therefore, nitrogen and carbon metabolisms play critical roles in the resistance of grapes against *B. cinerea* [26]. However, the main inhibitory action of antagonistic bacteria is to produce antifungal metabolites and antibiotics [27,28].

Given the wide-ranging and the importance of *B. cinerea* in agriculture, the control of GM is of great concern. In this context, our strategy was to develop new biotechnologies allowing grapevine to better resist parasitic pressures of *B. cinerea* through the isolation and screening of beneficial bacteria. Once screened, the mechanisms contributing to the biocontrol effect of selected bacteria was deciphered by determining their antimicrobial effect by in vivo test, and finally chlorophyll fluorescence imaging after *Botrytis* challenge. On the other hand, the in silico analysis of the whole genome of isolated strains has revealed the presence of “biocontrol-related” genes supporting their plant growth and biocontrol activities.

## 2. Materials and Methods

### 2.1. Soil Sampling

Soil samples were collected from different distinct locations of vineyard (Chardonnay cv.) in Meknes-Morocco. Sampling was carried out at the end of the growing season, after the occurrence of the asexual multiplication of *B. cinerea* and the appearance of symptoms of gray rot detectable on the grapevine. Although isolation of antagonistic bacterial strains was taken from soil samples of healthy grapevine, the bacteria coming from soil collected under the infected plants were selected as control, to compare the communities of both populations. At each sampling points, soil was collected aseptically from healthy and severely infected grapevines with *B. cinerea*. Samples were kept in paper bags placed in ice and processed within 24 h.

### 2.2. Isolation, Purification, and Enrichment of Antagonistic Bacteria 

The isolation of bacteria was carried out according to the protocol previously described by Nally et al. [29], with some modifications. Portions of 15 g of Rhizospheric soils were suspended in 250 mL of sterile Luria-Bertani (LB) liquid medium (tryptone 10 g/L; yeast extract 5 g/L; NaCl 10 g/L; pH 7.2). The enrichment culture was incubated on a rotary shaker (180 rpm) at 28 °C for 24 h. This operation was repeated for the soil recovered under the vines showing the GM symptoms. After shaking, the cultures were kept undisturbed for 30 min. Then, isolation of viable bacterial cultures from soil suspensions was done by serial dilution plate count in phosphate-buffer saline (PBS 10 mM, pH 6.5). Aliquots of 100 μL from the five different dilutions of the sequential enrichment were spread in triplicate on LB medium and incubated at 28 °C for 24–72 h until colony development. Colonies with distinct morphologies were picked and purified using the streaking method. The purified isolates were used to screen antagonistic bacteria against *B. cinerea*. Afterwards, pure bacterial cultures were maintained in cryovials containing LB broth with 25% glycerol and preserved at −80 °C.

### 2.3. In Vitro Screening of Potential Antagonistic Bacteria

Isolated strains were tested for antifungal activity against *B. cinerea*. Assays were performed by patching, in the middle of PDA medium (Sigma-Aldrich, MO, USA) plates, 5 mm of agar plug carrying freshly grown culture of the fungal pathogen. After that, a volume of 5 µL of suspension of each isolate was drooped at four sites approximately 1 cm from the rim of the plate and incubated at 22 °C for five days at which point we start measurements. Plates inoculated with *B. cinerea* were also used as control. The plates were visually inspected for the presence of inhibition zones between the fungus and the colonies considered to be potential antagonistic bacteria. The antifungal effect was estimated by calculating the percentage of inhibition (%) of mycelial growth measured as follows:

I(%) = (1 − Cn / Co) × 100(1)

where “Cn” is the average diameter of the mycelial in the presence of the antagonists and “Co” the average diameter of the control. Experiments were conducted in duplicate and the results reported are averages of three independent experiments. The diameter of the clear zones depends on the performance of the bacteria. Thus, the screened strains with high zones of clearing were selected for molecular identification and in vivo assays.

### 2.4. Identification of Antagonistic Bacteria

All potential antagonistic bacteria were identified by *16S rRNA* gene analysis. The *16S rRNA* gene was amplified by polymerase chain reaction (PCR) as previously described by [30]. Briefly, genomic DNA was extracted from a pure colony using the Wizard Genomic Purification DNA Kit (Promega Corp., Madison, WI, USA), according to the manufacturer’s instructions. Next, the bacterial 16S *rRNA* gene was amplified by PCR using FD2 (5- AGAGTTTGATCATGGCTCAG -3) and RP1(5- ACGGTTACCTTGTTACGACTT -3) primers. The PCR was carried out with a 50-µL final volume, containing 25-µL of Master Mix (Thermo Scientific Fermentas, Villebon sur Yvette, France), 2.5 µL of each primer, 15 µL sterile water, and 5 µL template DNA in a PTC-200 Thermocycler (C1000 touch thermal cycler, Bio-Rad, Hercules, CA, USA). The PCR conditions used were as follows: an initial denaturation step at 94 °C for 5 min, followed by 30 cycles of denaturation at 94 °C for 45 s, primer annealing at 55 °C for 45 s, and elongation at 72 °C for 1.5 min, and a final elongation step at 72 °C for 10 min.

The PCR product was subjected to electrophoresis on agarose gel with 0.5X TAE buffer (Tris Acetate-EDTA) at a ratio of 1% (weight/volume). Gels were stained with ethidium bromide, visualized under UV light (300 nm), and were then excised and purified using the Gene JET Gel Extraction Kit (Thermo Scientific Fermentas, Waltham, USA), as recommended by the manufacturer. A 1000-bp DNA ladder marker served as the standard size. The PCR product was commercially sequenced by Genewiz Co., Ltd. (Leipzig, Germany). The data output was analyzed, and the sequences were compared with sequences in the National Center for Biotechnology Information (NCBI) database using the BLAST search program (http://www.ncbi.nlm.nih.gov/). Alignment of *16S rRNA gene* sequences from GenBank database was performed using ClustalX 1.8.3 with default settings [31]. Phylogenesis was analyzed by MEGA version 7. Distances were calculated using the Kimura two parameter distance model. The tree was built by the neighbor-joining method. The dataset was boot- strapped 1000 times [32].

### 2.5. Biochemical Characterization of Biocontrol Isolates

Biochemical characteristics of isolated strains such as carbohydrate assimilation and fermentation were performed using BIOLOG GENIII microtiter plate (Hayward, CA, USA) as recommended by manufacturers.

### 2.6. Cellular Fatty Acid Analysis

Cellular fatty acid analysis was carried out at BCCM/LMG (the Belgian Co-ordinated Collections of Microorganisms, Ghent University, Ghent, Belgium). Bacterial isolates were grown for 24 h at 30 °C under aerobic conditions on LB medium. Inoculation and harvesting of the cells, and the extraction and analysis were performed conform to the recommendations of the commercial identification system MIDI (Microbial Identification System, Inc., Newark, DE, USA). The whole-cell fatty acid composition was determined gas chromatographically on an Agilent Technologies 6890N gas chromatograph (Santa Clara, CA, USA). The peak naming table MIDI TSBA 5.0 was used.

### 2.7. Whole Genome Sequencing, Assembly, and Annotation

The genomic DNA of strains was isolated using the Wizard Genomic Purification DNA Kit (Promega Corp., Madison, WI, USA) according to the manufacturer’s instructions. The integrity of extracted DNA was assessed by running the sample on 1% agarose gel. The genome sequence of the strain was sequenced at MicrobesNG (http://www.microbesng.uk) using the method summarized in Table S1. The draft genome sequences were used for annotation using the Rapid Annotation Subsystem Technology (RAST) server (http://rast.nmpdr.org) [33]. The annotated genes were analyzed using SEED database [34]. To predict the presence of secondary metabolites (SMs) gene clusters associated with biocontrol activity, the draft genome sequence was analyzed by antiSMASH software online (https://antismash.secondarymetabolites.org/#!/start) [35]. The Whole Genome Shotgun projects of the strains have been deposited in GenBank under the accession numbers JAFETM000000000 and JAFETL000000000. The version described in this paper version JAFETM010000000 and JAFETL010000000, respectively.

### 2.8. Grapevine In Vitro Plantlets

Plantlets of *Vitis vinifera* cv. Chardonnay (clone 7535) were micro-propagated by nodal explants grown on 15 mL of Murashige-Skoog (MS) agar medium in 25 mm-culture tubes as described by Ait Barka et al. [36]. Cultures were performed in a growth chamber under white fluorescent light (200 µmol/m^2^/s), with 16 h/8 h day/night photoperiod at a constant temperature of 26 °C.

### 2.9. Bacterial Isolates and Inoculum Preparation

Bacterial suspensions were prepared by inoculating 100 mL of Luria-Bertani (LB) liquid culture medium and incubated on a rotary shaker at 180 rpm at 28 °C for 18 h at which point they reached the late exponential growth phase. After incubation, cells were harvested by centrifugation at 4500× *g* at 4 °C for 15 min. Each culture was washed three times and resuspended in 20 mL of phosphate-buffered saline (PBS). The density of bacterial cultures was determined by spectrophotometry and adjusted at approximately 10^8^ colony-forming units (CFU) mL^−1^ with an optical density 0.8 at 600 nm (OD_600_).

For the inoculum preparation, the fungal pathogen, *B. cinerea* strain 630 was grown on potato dextrose agar (PDA) (Sigma-Aldrich, MO, USA) at 22 °C. The conidia were collected from 20-day-old culture plates by scratching the Petri dishes surface with sterile potato dextrose broth (PDB 24 g/L) medium and filtered to remove hyphae. Conidial concentrations were measured by a hemocytometer and the final density was adjusted to 10^5^ conidia/mL. After incubation during 3 h at 22 °C and 150 rpm, the resulting germinated spore suspension was used for plant inoculation.

### 2.10. Inoculation of In Vitro Plantlets with Antagonistic Bacteria and Infection by B. cinerea

Isolates with higher percentage of inhibition (%) during in vitro screening were tested for antagonistic activity against *B. cinerea* on sterile grapevine plantlets. Briefly, roots of 6-weeks-old grapevine plantlets were gently removed from the MS agar medium (Sigma-Aldrich, France) and transferred into magenta boxes containing 120 g of soil. Plantlets were then inoculated with bacterial inoculum at a concentration of 10^8^ CFU per g of soil. Control was treated with sterile PBS. Bacterized and non-bacterized plantlets were then grown for an additional 10 days. Thereafter, the upper side of each leaf was inoculated by a drop of 5 μL of *B. cinerea* germinated spore suspension. This protocol was used for measures of necrosis surfaces. The antifungal effect was estimated by calculating the percentage of inhibition (%) of mycelial growth measured as describes in in vitro tests.

For IMAGING-PAM analysis, plantlets were sprayed with the germinated spore suspension of *B. cinerea* to have a homogenous application. Plantlets were then placed in growth chamber at 22 °C. All the experiments were performed in triplicate.

### 2.11. IMAGING-PAM Analysis

Chlorophyll fluorescence parameters were measured with an IMAGING-PAM measuring system (Heinz Walz, Germany) using the saturation pulse method [37]. Control and bacterized plantlets were dark adapted for 30 min to establish the minimal fluorescence level (F0) and the maximal fluorescence (Fm) after a saturating flash (1 s; 13,000 µmol/m^2^/s). Each leaf was detached from the plantlet then exposed immediately to an actinic illumination of 79 µmol/m^2^/s. After fluorescence stabilization, a second saturating flash was imposed to determine the maximal fluorescence (Fm’) of light-adapted inflorescences. The effective PSII quantum yield, ΦPSII, is calculated according to the equation of Genty et al. [38]. The quantum yield of regulated energy dissipation in PSII, ΦNPQ, and the quantum yield of nonregulated energy dissipation in PSII, ΦNO, was calculated according to Kramer et al. (2004) [39]. Please note that ΦPSII + ΦNPQ + ΦNO = 1. The data were collected taking in consideration the entire leaf area including necrosis area. Measurements were taken 24 h before inoculation of antagonistic bacteria, 24 h before infection with *B. cinerea*, and 4 consecutive days after infection. The means ± standard deviations originated from three independent experiments realized in duplicates, each replicate consisted of four plantlets.

### 2.12. Statistical Analysis

The experimental design used was performed in triplicates. Statistical analyses were carried out using GraphPad Prism version 5.00 for Windows (GraphPad Software, San Diego, CA, USA) (www.graphpad.com). For lesion diameter Student’s *t*-tests (α > 0.05) was used to compare lesion area between inoculated and non-inoculated plants.

## 3. Results

### 3.1. Isolation and In Vitro Screening of Antagonistic Bacteria

**Figure 1:**
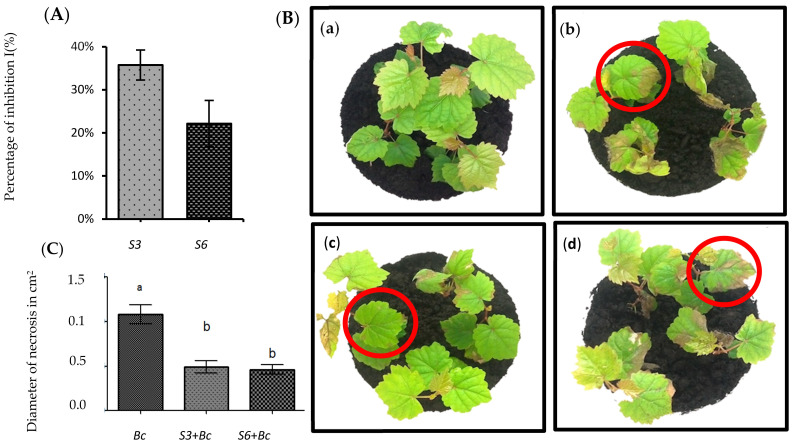
Impact of isolates on *B. cinerea* growth inhibition. Direct confrontation tests of isolates against *B. cinerea*. Results indicated are the mean of percent inhibition of mycelial growth of *B. cinerea* after 72 h of incubation in PDA medium (**A**). The Ability of isolate to protect grapevines (cv. Chardonnay), in planta, against *B. cinerea* (**B**,**C**). In vitro grapevine plantlets inoculated or not with S3 and S6, 72 hours post infection (hpi) with *B. cinerea* (**B**). (Control, (**a**); *B. cinerea*, (**b**); *S3* + *Bc*, (**c**); *S6* + *Bc*, (**d**)). Diameter of necrosis measured on leaves infected with *B. cinerea* (**C**).

As a result of multiple inoculations and purification, 42 pure cultures of potential antagonistic bacteria were successfully enriched and isolated from vineyard soil. These freshly isolated strains were purified on LB plates, selected based on their morphology, and used as objects of investigation. All isolates were screened for their ability to inhibit the mycelial growth of *B. cinerea* by direct confrontation tests in PDA medium plates. Out of 42 tested strains, only two isolates were estimated as antagonistic potential bacteria against this fungus. They were nominated S3 and S6 which were the most active against fungal culture, by showing the strong percentage of inhibition I (%) (Figure 1A). These isolates were tested for their ability to protect grapevines against GM *in planta*.

### 3.2. Disease Symptoms Were Significantly Reduced in Root-Bacterized Plantlets

To test the ability of *S3* and *S6* to protect grapevine in our system, we performed infection on whole potted-plant with *B. cinerea* strain 630 in control versus root-bacterized plantlets. Assays performed showed that the two antagonistic bacteria had high inhibiting ability against *B. cinerea* in grapevines three days after inoculation of the pathogen. They significantly reduced Botrytis-related necrosis by approximately 50% at 72 hpi (Figure 1B). In addition, disease symptoms were significantly less developed in bacterized plants, confirming the protective impact of *S3* and *S6* against *B. cinerea* (Figure 1C). Non-bacterized plantlets showed severe symptoms typical of GM, manifesting as necrosis around the infection spot (Figure 1C). In contrast, plants treated with the antagonistic bacteria exhibited a reduction in disease symptoms, displayed by a smaller size of necrosis diameter compared to the control. These isolates were selected for molecular investigation.

### 3.3. Identification of Antagonistic Bacteria

**Figure 2:**
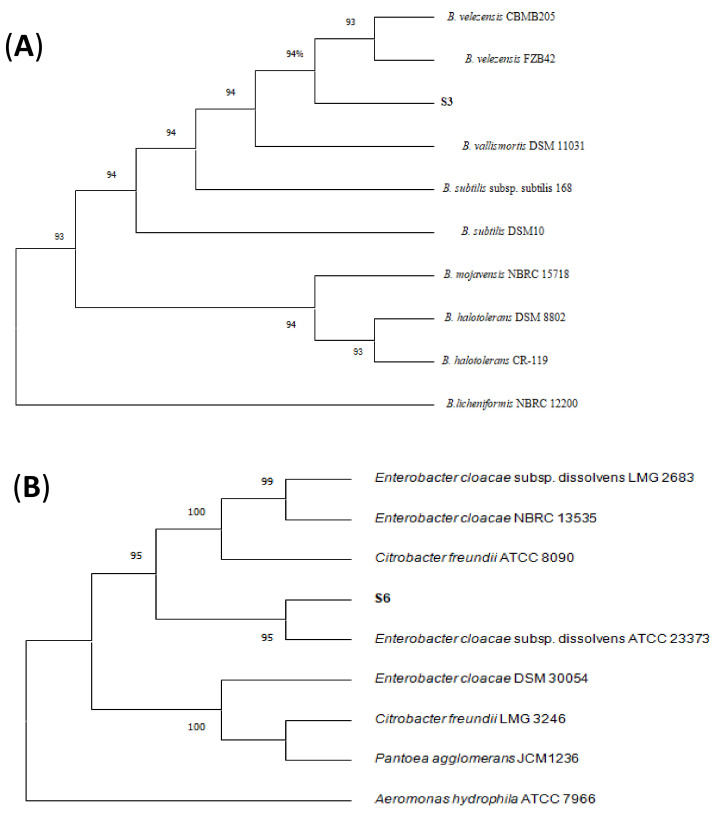
Neighbor-joining phylogenetic tree based on the 16SrRNA sequences of antagonistic isolates S3, and S6. showing the relationship with the genus *Bacillus* (**A**) and *Enterobacter* (**B**). The sequence of *B. licheniformis* NBRC 12200 and *Aeromonas hydrophila* ATCC 7966 were chosen as an out-group. Antagonistic strains are shown in bold.

The molecular identification using *16S rRNA* gene sequences of the antagonistic rhizobacteria show that *S3* and *S6* are closely related to *Bacillus velezensis* (100%), and *Enterobacter cloacae* (98.5%), respectively (Figure 2). Strains S3 and S6 also shared sequence identity with other species including *B. subtilis* (99%), and *Pantoea agglomerans* (98%), respectively (Figure 2). Both bacterial strains (S3 and S6) isolated under healthy canopy, were used for further characterization. In addition, to calculate the pair-wise average nucleotide identity (ANI) values of both strains with their closest known relatives, the draft genome sequences of strains S3 and S6 were compared against all type strain genomes available in the microbial genomes atlas (MiGA) webserver [40]. Results showed that strains S3 belongs to *Bacillus velezensis* (99% ANI). For strain S6, the closest relatives found was *Enterobacter cloacae* (98% ANI). Moreover, digital DDH values of both strains were compared against all type strain genomes available in the TYGS database [41]. The analysis generated DDH values more than 70% for both strains and the closet relatives were *Bacillus velezensis* and Enterobacter cloacae for strains S3 and S6, respectively.

### 3.4. Characterization of Biocontrol Isolates

The data presented in Table 1 showed some biochemical characteristics of isolated strains. The selected antagonistic isolates (S3 and S6) were characterized by biochemical methods. The optimum growth conditions of isolates are at 28°C, pH 6.0, and in the presence of 1% NaCl. Although cells of strain S3 were sensitive to fusidic acid, minocycline, naldixic acid, rifamycin SV, lncomycin, and vancomycin, strain S6 was resistant to them. Isolates were able to grow in the presence of sodium butyrate, guanidine HCl, lithium chloride, potassium tellurite, and tetrazolium violet, but did not grow in the presence of sodium bromate, D-serine, and niaproof 4 except *E. cloacae*, which was resistant (Table 1). The two isolates failed to hydrolyze gelatin. Furthermore, the strains showed different abilities to use different carbon sources (Table 1). They were able to assimilate, D-cellobiose, mannose, mannitol, and N-acetyl-glucosamine, L-glutamic acid, sucrose, L-aspartic acid, D-maltose, D-fucose (except *B. velezensis S3*), and L-histidine, as sole carbon sources whereas, 3-methylglucose, α-ketobutyric acid, and D-aspartic acid were not used by both strains. The isolates exhibited different patterns of cellular fatty acids profile features characterized by different level of C15: 0 iso-anteiso, C17: 0 iso-anteiso, summed feature 2 (comprising C12: 0 aldehyde, C14: 0 3-OH/ C16: 1 iso I and/or unknown ECL 10.928), C18:1 ω7c, C16: 0, C17:0 cyclo, C14:0, summed feature 3 (comprising C16:1 ω7c/15 iso 2OH), C12: 0, and C19: 0 cyclo ω8c. *Isolate S3* was characterized by a fatty acid profile dominated to an unusual extent (> 98%) by saturated fatty acids (Figure S1). Hence, cells had less iso odd-numbered fatty acid and more anteiso odd-numbered fatty acid, with the major fatty acid being anteiso-C15:0(37.18%) (Table 1). Cells of strain S6 exhibited only 37,88% of saturated fatty acids but revealed other structure as branched chain (29,51%), cyclopropane (16,90%), and hydroxy unsaturated (14.68%) fatty acids that was deficient in strain S3 (Figure S2). The major fatty acid for *S6* were C16:0 (18.07%) (Table 2).

### 3.5. Genomic Feature and In Silico Analysis

As summarized in Table 3, the draft genome sequence of the strain S3, assembled into 21 contigs, consists of 4157, 680p with a 46.35% G+C. Strain S6, grouped into 30 contigs exhibited genome size of 4, 604, 658bp, and the GC content was 55.90%. The total number of predicted protein-coding sequences and RNAs was 4316 and 98 of RNAs for S3 versus 4228 and 116 of RNAs in S6, respectively. The genome characteristics of each isolate were detailed in Table 3. The predicted coding sequences (CDS) of S6 were classified into 560 subsystems, most of which were involved in amino acids and derivatives synthesis, carbohydrate and protein metabolism, cofactors, vitamins, prosthetic groups and pigment formations, and stress response (Figure S2). Based on phylogenic analysis, chemical characteristics, and genotypic data described in this report, the isolate S3 belongs to *B. velezensis* while S6 is attributed to *E. cloacae* species. The draft genome sequence of each isolate was subject to multiple genomic analyses with the aim to identify all the genes potentially responsible for its antimicrobial activity especially those produced by non-ribosomal peptides synthetases (NRPSs). The in silico analysis using antiSMASH server revealed the presence of different secondary metabolites gene clusters (SMGCs) involved in biocontrol. The draft genome of S3 predicted the presence of many SMGCs, including bacillibactin, fengycin, surfactin and bacillaene. In our analysis of S6 genome, we detected the presence of non-ribosomal peptide (NRPs) and bacteriocin.

### 3.6. Antagonistic Strains Prevent Plants from Considerable Photo-Inhibition of PSII after Pathogen Challenge

**Figure 3:**
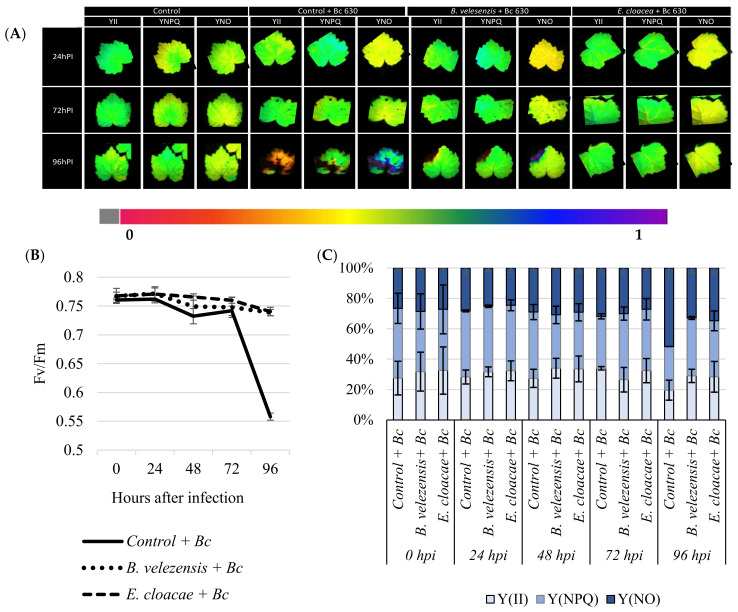
*B. velezensis S3* and *E. cloacae S6* prevent grapevines from PSII photo-inhibition four days after infection with *B. cinerea*. Images of the effective PSII quantum yield Y(II), the quantum yield of regulated energy dissipation Y(NPQ) and of nonregulated energy dissipation Y(NO), from grapevine leaves inoculated or not with *B. velezensis S3* and *E. cloacae S6* 0, 24, 48, 72, and 96 hpi with *B. cinerea*. The pixel value display is based on a false-color scale ranging from black (0.000) via red, yellow, green, blue, to purple (ending at 1.00). The figure shows representative images of one from two independent experiments (**A**). Evolution of the maximum PSII quantum yield (Fv/Fm) from grapevine leaves inoculated or not with *B. velezensis S3* and *E. cloacae S6* 0, 24, 48, 72, and 96 hpi with *B. cinerea* (**B**). Changes in chlorophyll fluorescence parameters (Y(II), Y(NO), and Y(NPQ)) from grapevine leaves inoculated or not with *B. velezensis S3* and *E. cloacae S6* 0, 24, 48, 72, and 96 hpi with *B. cinerea*. Excitation flux at PSII in infected leaves (**C**). Values shown are means ± SD of two independent repetitions (each repetition was realized in triplicates).

To evaluate the effect of root inoculation with *B. velezensis* S3 and *E. cloacae* S6 on photosynthesis before and 24, 48, 72, and 96 h post-infection with *B. cinerea*, changes in excitation flux at PSII were monitored. Photosynthetic parameters including effective PSII quantum yield Y(II), quantum yield of nonregulated energy dissipation Y(NO), quantum yield of regulated energy dissipation Y(NPQ), and maximum PSII quantum yield (Fv/Fm) were evaluated. The false-color scales shown at the bottom of the fluorescence images indicate the amplitude of the particular parameter (Figure 3a). Before infection with the pathogen, no significant difference between bacterized and non-bacterized plantlets was observed regarding the monitored photosynthetic parameters. The value Fv/Fm were around 0.8 before the infection (Figure 3b). Furthermore, no significant fluctuation was occurred in Fv/Fm value in plantlets during 3 days after infection. However, considerable PSII photo-inhibition was observed when plants were not treated with bacteria and were exposed to a prolonged infection with B. cinerea (Figure 3b), while at the same time, in response to *B. cinerea*, bacterized plantlets exhibit indistinct symptoms (Figure 3a), and Fv/Fm value remain constant during kinetics (Figure 3b). Best results were displayed by the two strains. In the case of bacterized plantlets, the higher effective quantum yield of photochemical energy conversion in PSII Y(II) was maintained before as well at 96 hpi with *B. cinerea* in comparison to the non-bacterized plant, which decreased. The quantum yield of regulated energy n PSII Y(NPQ) was down-regulated before infection, compared to control. In contrast, four days after infection, the bacterized plantlets dissipated a higher Y(NPQ) than control (Figure 3c). Although no difference in the quantum yield of nonregulated energy loss in PSII Y(NO) was observed between plantlets before infection, this response resulted in a lower Y(NO) in bacterized plantlets after a prolonged infection with *B. cinerea*.

## 4. Discussion

Rhizosphere is an extremely competitive environment, where diverse genera of microorganisms are constantly competing for resources and with each other to survive [42]. This work was undertaken to screen efficient competitive strains from vineyard to control grapevine GM. Among 42, two strains (S3 and S6) have been showed the best performance against the *B. cinerea*. When plantlets were previously bacterized with S3 or S6 then infected with the *B. cinerea*, the symptoms of gray mold were reduced compared to control, confirming therefore the beneficial effect of these strains as reported in in vitro confrontation test. Similar results were reported by Miotto-Vilanova et al., [37] when grapevine plantlets were previously bacterized with *Burkholderia phytofirmans* PsJN before infection with *B. cinerea*.

The *16S rRNA* gene sequences of S3 exhibited 100% similarity to *B. velezensis*, while the strain S6 showed 98.5% similarity to *E. cloacae. B. velezensis* and *E. cloacae* have been frequently reported as plant growth promoting bacteria and/or biocontrol agents [43,44,45,46,47,48,49,50]. The fact that many *Bacilus* species have very close phenotypic and physiological properties as well as *16S rRNAs* gene sequences makes their classification very difficult [51]. The *Bacillus* genus encompasses a large genetic biodiversity [52], and in addition to the “original members” *B. subtilis*, *B. licheniformis* and *B. pumilus*, earlier described by Gordon et al. [53], many novel species belonging to the *B. subtilis* species complex have been described in recent decades, among them *B amyloliquefaciens* [54], *B. velezensis* [55], and *B. methylotrophicus* [56]. Recently, these *Bacillus* species have been reclassified by genome comparisons and phylogenomic analyses [57]. In particular, *B. methylotrophicus* and *B. amyloliquefaciens subsp. plantarum* were later confirmed as heterotypic synonyms of *B. velezensis* [58]. Hence, many studies have suggested that *B. amyloliquefaciens* subsp. *Plantarum, B. methylotrophicus*, and *B. velezensis* formed a monophyletic group [59,60].

The plant growth-promoting ability has been related to distinctive physiological activities and molecular changes that might have an intense impact on the fitness (growth and/or health) of plants. Both *B. velezensis* S3 and *E. cloacae* S6 have triggered the resistance of grapevine toward GM disease. *B. velezensis* is a heterotypic synonym of *B. methylotrophicus*, *B. amyloliquefaciens subsp. plantarum*, and *B. oryzicola,* and is used to control plant fungal diseases [61] such as *B. cinerea* [62,63,64]. *Bacillus* species are promising agent for the biological control of postharvest diseases [52], particularly *B. velezensis,* which is widely used in agriculture [65]. Additionally, Morales-Cedeño et al. [66] showed that *B. velezensis* BLE7 showed similar activities to thiabendazole, a commonly used fungicide for *B. cinerea* [67]. In cells of *B. velezensis*, the composition of iso and anteiso fatty acids was higher. The a-C15:0 became even more prominent component of the fatty acids. Furthermore, the major changes observed were a sharp decrease in a-C17:0 content in parallel with a significant increase in a-C15:0. The shift to a fatty acid profile dominated by a-C15:0 draws attention to the critical role of this fatty acid in low temperatures (4 °C) growth [68], presumably due to its physical properties and their effects in maintaining a fluid, liquid-crystalline state of membrane lipids [69], making this bacterium a potential effective biocontrol agent in extreme environments.

*Enterobacter cloacae* is perhaps best known as an opportunistic human pathogen that is commonly found in hospitals causing a wide range of infections, although some lineages have also been described as plant endophytes [49]. Indeed, several *Enterobacter* species can colonize internal plant tissues, improve plant growth and prevent from pathogens attacks [70,71,72,73,74]. Thus, *Entrobacter cloacae* was found to halt fungal phytopathogens growth such as *Phytium debaryanum* by 35.13% and *Rhizoctonia solani* with pathogen growth inhibition up to 60% [75]. Additionally, *E. cloacae* inhibited the development of *Pythium myriotyum*, *Gaeumannomyces graminis* and *Heterobasidion annosum* [52]. 

The observed inhibition is due to production of several antifungal metabolites such as H_2_S, ammonia and volatile compounds such as phenylethyl alcohol, 4,5-dimethy l-1-hexene, and butyl acetate that halt the growth of fungal phytopathogens [50,76]. Recently, it has been reported that *E. cloacae* is able to produce inorganic volatile substances such as ammonia, IAA and hydroxamate siderophore, hydrogen cyanide and salicylic acid [74,77], in addition to chitinase, cellulase, and beta-glucosidase enzyme all of which may participate to the biocontrol activity [73,78,79,80]. Furthermore, Chaouachi et al. [81] reported for the first time volatile organic compounds (VOCs) with antifungal activity produced by *E. cloacae* against *B. cinerea* decay on tomato fruit. Similarly, *Bacillus* species such as *B. velezensis* [63,82] are known to produce antifungal VOCs against several phytopathogens including *B. cinerea*.

In addition, Patel et al. [83] and Agbodjato et al. [84] reported that AIA with ammonia production prevents the development of various plant pathogenic fungi and enhance the plant growth. Additionally, solubilization of phosphate and potassium occurs due to the production of protons and oxalic, tartaric acid and polysaccharidic capsules by bacteria [76,85]. Romero et al. [86] have reported that antimicrobial compounds produced by *Bacillus* spp. are mainly classified into two categories: ribosome-synthesized peptides such as bacteriocin, and small microbial peptides enzymatically synthesized by non-ribosomal pathways, mainly cyclic lipopeptides (CLPs). Since *B. velezensis* is not pathogenic to humans, different strains of *B. velezensis*, which is a typical PGPR, have received significant attention in the last decade.

So far, *B. velezensis* was described to halt the growth of many pathogenic fungi, such as *Aspergillus flavus* [87], *Cylindrocladium quinqueseptatum*, *Cryphonectria parasitica* and *Helicobasidium purpureum* [88], *Fusarium oxysporum* and *Ralstonia solanacearum* [89], and *Rhizoctonia solani* AG1-IB [90], by the biosynthesis of β-1,3-1,4-glucanase, lipopeptide antibiotics (surfactin, iturin, and fengycin, for example), polyketides (actinomycin D, bacitracin, and cyclosporin A, for example), siderophores, and NH3 [44,91,92,93]. These data are in line with our in silico analysis of both bacteria *B. velezensis* S3 and *E. cloacae* S6. Thus, analysis of *E. cloacae* S6 genome pointed out the presence of non-ribosomal peptide synthetase (NRPS) and bacteriocin, while the genome of strain *B. velezensis* S3 revealed different SMGCs, such as bacillibactin, fengycin, surfactin and bacillaene. Hence, it was hypothesized that these strains might produce a variety of antifungal compounds that participate in the control of the grapevine GM disease.

The photosynthesis provides near 90–95% of plants dry matter and the metabolic energy needed for plant’s development. Pathogen attack not only impact plant defenses reactions but can also lead to changes in the rate of photosynthesis and therefore the carbohydrates metabolism [37]. Hence, four days after infection, it appears that under *Botrytis* exposure, thermal dissipation in control was down-regulated not due to an increased PSII quantum efficiency, but due to an increased nonregulated energy loss in PSII, suggesting that both photochemical energy conversion and protective regulatory mechanism were insufficient [94]. Consequently, the larger portion of absorbed light energy is allocated to nonregulated energy loss in PSII [95]. The latter parameter indicates an irreversible damage of photosynthetic apparatus as confirmed by the decreased Fv/Fm ratio, since the Y(NO) leads to the formation of singlet oxygen via the triplet state of chlorophyll (3chl*) [96,97]. In line with our results, several reports on photosynthesis have indicated that photosynthesis rates are altered after infection with several plant pathogens [4,95]. We also have observed that the regulation of mechanisms involved in nonphotochemical dissipation of energy was blocked, making grapevine plantlets unable to protect themselves against damage from excessed illumination [37]. A significant decline of effective quantum yield of PSII(Y(II)) complemented by a quantum yield of regulated energy dissipation nonphotochemical chlorophyll fluorescence quenching [Y(NPQ)] increase was observed after the *B. cinerea* infection. The [Y(NPQ)] is a molecular adaptation that represents the fastest response of the photosynthetic membrane to excess light. It is a protective process in which excess absorbed light energy is dissipated into heat [98,99] and prevent the photosynthetic apparatus from oxidative damage [100]. However, the irreversible damages described above were prevented/attenuated when grapevine plantlets were bacterized either with *B. velezensis S3* and *E. cloacae S6*, probably by restricting mycelial development, and therefore protecting photosynthesis apparatus. Therefore, finding safe and eco-friendly alternatives to synthetic fungicides is urgently needed to control postharvest diseases of fruit [101]. Hence, this study provided new biotechnologies by reporting for the first time that following root inoculation, *B. velezensis S3* and *E. cloacae S6* allowing grapevine to better resist aerial parasitic pressures of *B. cinerea* while at the same time prevent grapevine from considerable photo-inhibition.

## 5. Conclusions

In conclusion, both bacteria screened as efficient anti-Botrytis were identified as *B. velezensis* S3 and *E. cloacae* S6. In silico analysis of draft genome sequences of *B. velezensis S3* indicates the presence of gene clusters involved in amino acids and derivatives synthesis, carbohydrates and proteins metabolism, cofactors, vitamins, prosthetic groups and pigment formations, and stress response; while that of the *E. cloacae S6* revealed the presence of different secondary metabolites gene clusters, including bacillibactin, fengycin, surfactin and bacillaene and the presence of non-ribosomal peptide synthetase and bacteriocin.

Further work is obviously required to characterize how these bacteria trigger the biotic stress resistance in plants to establish a set of biotic stress biomarkers that might help to predict efficacy of induced resistance for different crops. In addition, it would also be very interesting to analyze the plant’s response to colonization, i.e., lipid content, fatty acid composition as well as secondary metabolites profiles.

Nevertheless, genus *Enterobacter* is a member of the ESKAPE group, which contains the major opportunistic and multi-resistant bacterial pathogens for humans during recent decades in hospital wards. Therefore, a deeper progress in genome sequencing of *E. cloacae* S6 is critical before a potential use of this strain for plant protection. Additionally, deciphering the mechanisms of horizontal gene transfer that may occur in specific microhabitats may be a key step in the development of a regulatory framework for the environmental release of these bacteria.

## Figures and Tables

**Table 1:** Biochemical characteristics for the antagonistic strains based on BIOLOG GENIII microtiter plate (Hayward, CA, USA).

	**Oxidation of**			**Oxidation of**			**Oxidation of**	
	**S6**	**S3**		**S6**	**S3**		**S6**	**S3**
3-methylglucose	−	−	D-melibiose	+	+	L-lacticacid	+	+
Aceticacid	+	−	D-raffinose	+	−	L-malicacid	+	+
acetoaceticacid	−	−	D-saccharicacid	+	±	L-pyroglutamicacid	±	±
bromo-succinicacid	+	−	D-salicin	±	−	L-rhamnose	+	−
Citricacid	+	±	D-serine	−	−	L-serine	+	−
D-arabitol	±	−	D-sorbitol	+	+	Methylpyruvate	+	±
D-asparticacid	−	−	D-trehalose	+	+	mucicacid	+	±
D-cellobiose	+	+	D-turanose	±	±	myo-inositol	+	±
Dextrin	±	+	Formicacid	±	−	N-acetylneuraminicacid	−	±
D-fructose	+	+	Gelatin	−	−	N-acetyl-D-galactosamine	+	±
D-fructose-6-PO4	+	±	Gentiobiose	+	−	N-acetyl-D-glucosamine	+	+
D-fucose	±	−	Glucuronamide	+	+	N-acetyl-β-D-mannosamine	+	+
D-galactose	+	−	Glycerol	+	±	Pectin	±	±
D-galacturonicacid	+	+	Glycyl-L-proline	+	−	p-hydroxyphenylaceticacid	±	−
D-gluconicacid	+	+	Inosine	+	±	propionicacid	−	−
D-glucose-6-PO4	+	±	L-alanine	+	+	Quinicacid	−	±
D-glucuronicacid	+	+	L-arginine	±	+	Stachyose	+	−
D-lacticacidmethylester	±	±	L-asparticacid	+	+	Sucrose	+	+
D-malicacid	−	−	L-fucose	±	−	Tween 40	−	±
D-maltose	+	+	L-galactonicacid lactone	+	+	A-D-glucose	+	+
D-mannitol	+	+	L-glutamicacid	+	+			
D-mannose	+	+	L-histidine	+	+			
**Growth in the presence of**	**S6**	**S3**	**Growth in the presence of**	**S6**	**S3**	**Growth in the presence of**	**S6**	**S3**
1% Nacl	+	+	Lincomycin	+	−	Rifamycin SV	+	−
4% Nacl	+	+	Lithiumchloride	+	+	Sodium bromate	−	−
8% Nacl	±	±	Minocycline	±	−	Sodium butyrate	+	+
1% sodium lactate	+	+	Nalidixicacid	±	−	Tetrazoliumblue	+	−
Aztreonam	±	−	Niaproof 4	+	−	Tetrazolium violet	+	±
D-serine	−	−	pH 5	±	−	Troleandomycin	+	−
Fusidicacid	±	−	pH 6	+	+	Vancomycin	+	−
Guanidinehcl	+	+	Potassium tellurite	+	+			

**Table 2:** Major cellular fatty acid content of S3 and S6 strains.

Structure	Fatty Acid	Systematic Name Saturated	% In Isolated Strains	
			S3	S6
Saturated	C12: 0	Dodecanoic	4.23	7.52
	C13: 0	Tridecanoic	-	1.68
	C13: 0 ANTEISO		0.40	-
	C14: 0	Tetradecanoic	1.01	8.23
	C14: 0 ISO		1,11	-
	C15: 0 ISO		11.95	-
	C15: 0 ANTEISO		37.18	-
	C16: 0	Hexadecanoic	17.16	18.07
	C16: 0 ISO		2.41	-
	C17: 0	Heptadecanoic	0.57	2.38
	C17: 0 ISO		9.44	-
	C17: 0 ANTEISO		11.64	-
	C18: 0	Octadecenoic	1.21	-
Hydroxy Unsaturated	C15: 0 3-OH	3- Hydroxy- pentadecenoic	-	0.58
	C16: 1 ω 5c	cis-11-Hexadecenoic	-	-
	C16: 1 ω 11c		1.69	-
	C17:1 ω 8c		-	0.60
	C18: 1 ω 7c	cis-11- Octadecenoic	-	13.49
Cyclopropane	C17: 0 cyclo	Cyclo-heptadecanoic	-	15.04
	C19: 0 cyclo ω 8c	9-(2-eptylcyclopropyl) Nonanoic	-	1.86
Branched chain	Summed feature 1	C 15:1 ISO H/ C 13:0 3OH C 15:1 ISO I/ C 13:0 3OH	-	3.28
	Summed feature 2	C12: 0 aldehyde, C 16:1 ISO I/ C 14:0 3OH and/or unknown ECL 10.928	-	17.85
	Summed feature 3	C 16:1 ω7c/ 15 iso 2OH	-	8.38

**Table 3:** Genomic future and in silico analysis of draft genome sequences of bacterial isolates.

Attribute	S3	S6
Size (bp)	4.15 Mb	4.60 Mb
G+C content (%)	46.35	55.90
RNA genes	98	101
Protein-coding genes	3983	4258
N50	959,830	319,447
L50	2	4
Number of Subsystems	328	560
Most frequently species	*B. velezensis*	*E. cloacae*
Number of contigs	21	30

## Supplementary Materials

Supplementary materials can be found at https://www.mdpi.com/article/10.3390/microorganisms9071386/s1. Table S1. Project information of the draft genome sequences of the strains. Figure S1. Proportion of different structure in a fatty acid profile of isolated strains. Figure S2. Subsystem information of the strain *B. velezensis* S3 and *E. cloacae* S6 and predicted by SEED viewer, most of which were involved in amino acids and derivatives synthesis, carbohydrate metabolism, cofactors, vitamins, prosthetic groups and pigment formations, and stress response.
