# Supplementary material for: Beneficial Microorganisms to Control the Gray Mold of Grapevine: From Screening to Mechanisms
Source: Microorganisms. 2021 Jun 25;9(7):1386. doi: 10.3390/microorganisms9071386 (PMC8304954; doi:10.3390/microorganisms9071386)
Supplement: Supplementary file 1 [file microorganisms-09-01386-s001.zip › microorganisms-1266926-supplementary.pdf]

microorganisms

Supplementary Materials

Table S1. Project information of the draft genome sequences of the strains.

Property	Strain S3 Genome	Strain S6 Genome
Sequencing platforms	Illumina MiSeq, HiSeq 2500	Illumina MiSeq, HiSeq 2500
Libraries used	2- 250-bp	2- 250-bp
Mean coverage	209X	155X
Assembly Method	SPAdes v. 3.9.0	SPAdes v. 3.9.0
BIOPROJECT	PRJNA700080	PRJNA700080
BioSample	SAMN17823213	SAMN17823214
Source Material Identifier	Soil and grapes samples	Soil and grapes samples
Project relevance	Agriculture	Agriculture
GenBank ID	JAFETM000000000	JAFETL000000000

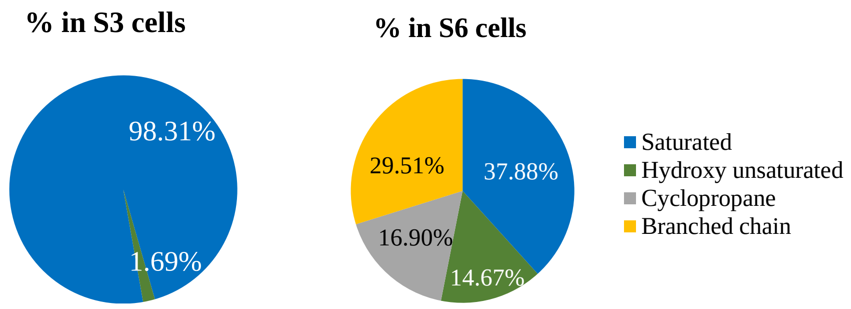

Figure S1. Proportion of different structure in a fatty acid profile of isolated strains.

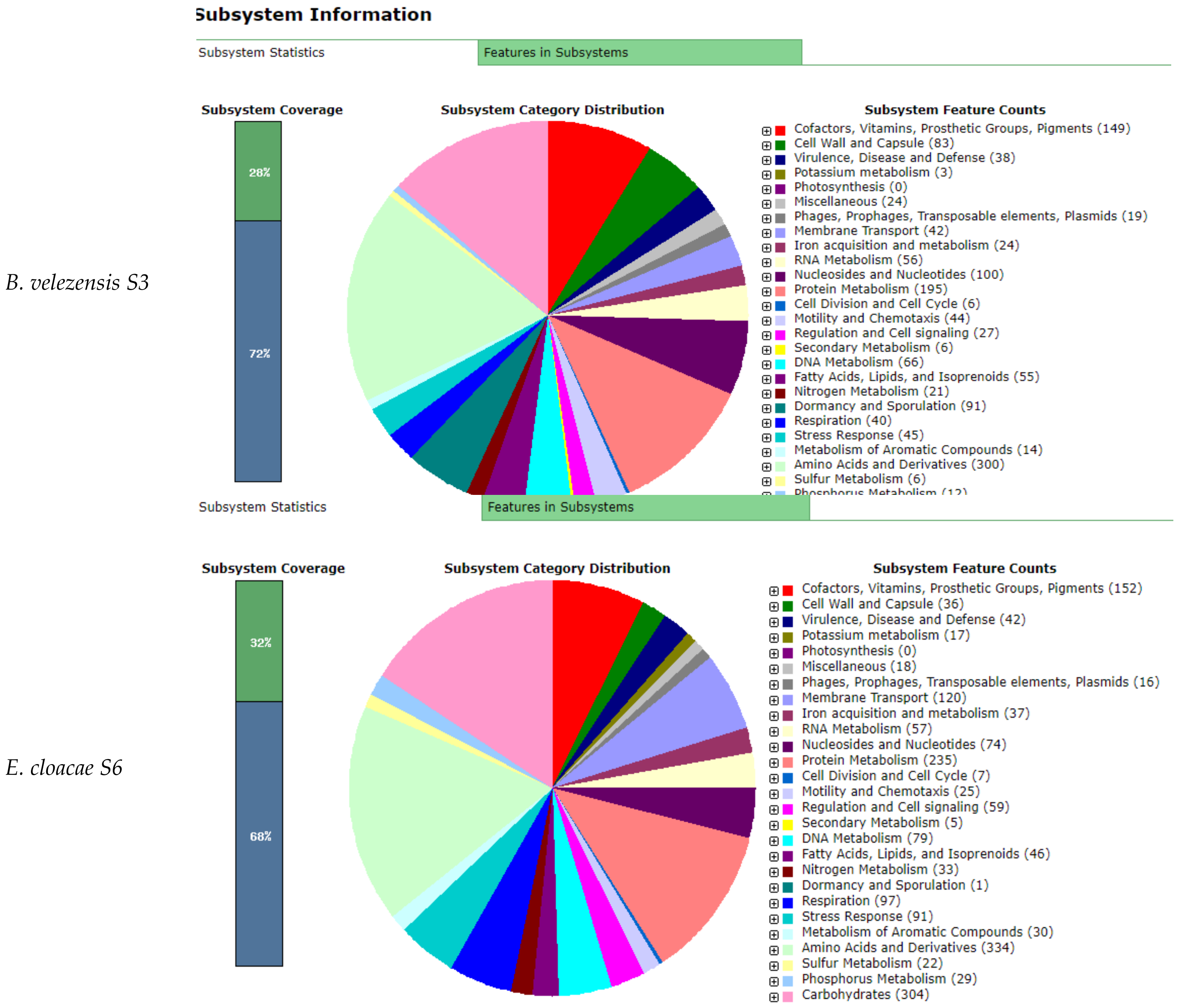

Figure S2. Subsystem information of the strain *B. velezensis* S3 and *E. cloacae* S6 and predicted by SEED viewer, most of which were involved in amino acids and derivatives synthesis, carbohydrate metabolism, cofactors, vitamins, prosthetic groups and pigment formations, and stress response.
